# Interleukin-6 trans signalling enhances photodynamic therapy by modulating cell cycling

**DOI:** 10.1038/sj.bjc.6604073

**Published:** 2007-11-06

**Authors:** L-H Wei, H Baumann, E Tracy, Y Wang, A Hutson, S Rose-John, B W Henderson

**Affiliations:** 1 Department of Oncology, National Taiwan University Hospital, No. 7, Chung-Shan South Road, Taipei 100, Taiwan; 2 Department of Molecular and Cellular Biology, Roswell Park Cancer Institute, Buffalo, NY 14263, USA; 3 Department of Biostatistics, Roswell Park Cancer Institute, Buffalo, NY 14263, USA; 4 Kiel Institut für Biochemie, Christian-Albrechts-Universität, Kiel, Germany; 5 Department of Cell Stress Biology and PDT Center, Roswell Park Cancer Institute, Buffalo, NY 14263, USA

**Keywords:** cell cycle, interleukin-6, photodynamic therapy, soluble interleukin-6 receptor

## Abstract

Photodynamic therapy (PDT) of solid tumours causes tissue damage that elicits local and systemic inflammation with major involvement of interleukin-6 (IL-6). We have previously reported that PDT-treated cells lose responsiveness to IL-6 cytokines. Therefore, it is unclear whether PDT surviving tumour cells are subject to regulation by IL-6 and whether this regulation could contribute to tumour control by PDT. We demonstrate in epithelial tumour cells that while the action of IL-6 cytokines through their membrane receptors is attenuated, regulation by IL-6 via trans-signalling is established. Soluble interleukin-6 receptor-*α* (IL-6R*α*) (sIL-6R*α*) and IL-6 were released by leucocytes in the presence of conditioned medium from PDT-treated tumour cells. Cells that had lost their membrane receptor IL-6R*α* due to PDT responded to treatment with the IL-6R–IL-6 complex (Hyper-IL-6) with activation of signal transducers and activator of transcription (STAT3) and ERK. Photodynamic therapy-treated cells, which were maintained during post-PDT recovery in presence of IL-6 or Hyper-IL-6, showed an enhanced suppression of proliferation. Cytokine-dependent inhibition of proliferation correlated with a decrease in cyclin E, CDK2 and Cdc25A, and enhancement of p27kip1 and hypophosphorylated Rb. The IL-6 trans-signalling-mediated attenuation of cell proliferation was also effective *in vivo* detectable by an improved Colon26 tumour cure by PDT combined with Hyper-IL-6 treatment. Prevention of IL-6 trans-signalling using soluble gp130 reduced curability. The data suggest that the post-PDT tumour milieu contains the necessary components to establish effective IL-6 trans-signalling, thus providing a means for more effective tumour control.

Photodynamic therapy (PDT) is an effective therapy for local malignant tumours, which uses the activation of tumour-localising photosensitising agents by visible light (Henderson and Gollnick, 2003). It results in the local generation of cytotoxic singlet oxygen and other reactive oxygen species, setting in motion a sequence of photochemical and photobiological processes that can cause irreversible oxidative damage to cells of the tumour parenchyma and stroma (Girotti, 2001). The initiating event is the immediate oxidative modification of lipids and proteins, followed by cell signalling events that over minutes to hours result in changes in gene expression (Luna *et al*, 1994; Kick *et al*, 1996), stress responses, repair reactions and/or activation of cell death mechanisms (Ahmad *et al*, 1998; Wong *et al*, 2003; Zhuang *et al*, 2003). In particular, photo-oxidation of plasma membrane lipids leads to accelerated phospholipid degradation and generation of potent inflammatory mediators (Korbelik, 1996; Girotti, 2001). Increased expression of specific cyto- and chemokines, and vascular adhesion molecules creates a tumour milieu that within hours attracts host inflammatory immune cells, neutrophils and macrophages (Gollnick *et al*, 1997, 2003). Inflammation has been identified as an important contributor to long-term tumour control by PDT (de Vree *et al*, 1996; Henderson *et al*, 2004) through stimulation of an antitumour immune response (Gollnick *et al*, 2006; Korbelik *et al*, 1996a, 1996b).

Interleukin-6 (IL-6) mRNA and protein are upregulated in certain tumour cells after PDT *in vitro* and IL-6 is generally the most markedly enhanced cytokine in tumour tissues after PDT *in vivo* (Kick *et al*, 1995; Gollnick *et al*, 1997). The biological activities of IL-6 are classically mediated through binding to a membrane-bound cognate receptor interleukin-6 receptor-*α* (IL-6R*α*) (gp80) and triggering of a signal transducer, gp130 (Kishimoto *et al*, 1995; Hibi *et al*, 1996). Upon activation, gp130-derived signals alter many cellular functions, such as cell proliferation and survival, differentiation, mobility and angiogenesis (Hirano *et al*, 2000; Wei *et al*, 2003).

We have reported earlier that PDT can abolish cellular responsiveness to cytokines and growth factors, including IL-6, through the loss of their respective membrane receptors, which would render the abundantly present IL-6 in the tumour environment ineffective (Wong *et al*, 2003). However, many of the biological activities assigned to IL-6 can also be mediated via a soluble form of the cognate IL-6 receptor-*α* (sIL-6R*α*) during acute inflammation. The soluble IL-6R can be generated by two distinct mechanisms, namely, by proteolytic cleavage of the membrane receptor or by translation of a differentially spliced mRNA (Rose-John and Heinrich, 1994). The sIL-6R*α* forms an agonistic complex with IL-6 and binds membrane gp130 to trigger cellular responses in a process termed IL-6 trans-signalling (Rose-John and Heinrich, 1994). We hypothesise that IL-6 trans-signalling can compensate in part for PDT-induced loss of signalling through the membrane-bound IL-6R*α*, thus providing IL-6 responsiveness in the post-PDT tumour environment. Our current study demonstrates for the first time that significant amounts of sIL-6R*α*, capable of mediating the effects of PDT-induced IL-6, can be expected to be generated in the tumour environment. They further show that this enhances tumour control by PDT.

## MATERIALS AND METHODS

### Cell culture

Tumour cell lines were obtained from the American Type Culture Collection (Rockville, MD, USA). Human cervical carcinoma cell lines HeLa and C33A were maintained in DMEM supplemented with 2 mM L-glutamine, 1% streptomycin–penicillin, gentamicin (100 *μ*g ml^−1^) and 10% foetal bovine serum (FBS). Murine Colon26 (colon carcinoma) cells were maintained in RPMI-1640 medium with 10% FBS and 1% streptomycin–penicillin. Human macrophages were obtained from the lung parenchyma of patients undergoing thoracic surgery (Loewen *et al*. 2005).

### Animals and tumour system

Colon26 tumours were propagated in pathogen-free BALB/cJ mice (Jackson Laboratory, Bar Harbor, ME, USA). Animals were housed in microisolator cages in a laminar flow unit under ambient light. Six- to 12-week-old animals were inoculated intradermally on the right and/or left shoulder with 10^6^ tumour cells harvested from exponentially growing cultures. Tumours were used for experimentation ∼10 days after inoculation when they had reached 6–8 mm in diameter. The RPCI Institutional Animal Care and Use Committee approved all of the procedures carried out in this study.

### Photosensitiser

Clinical-grade, pyrogen-free 2-[1-hexyloxyethyl]-2-devinyl pyropheophorbide-a (HPPH) was obtained from the Roswell Park Pharmacy (Buffalo, NY, USA) and reconstituted to 0.4 mM in pyrogen-free 5% dextrose containing 2% ethanol and 0.1% Tween.

### Photodynamic therapy treatment

For *in vitro* experiments, cells at subconfluence were incubated in the dark at 37°C for 4 h with medium containing HPPH doses as indicated in the text. Medium was replaced by HPPH-free medium and the cells were exposed to 665 nm laser light at a fluence of 1 or 2 J cm^−2^ and a fluence rate of 7 or 14 mW cm^−2^ (see description of individual experiments).

For *in vivo* experiments, animals were injected via tail vein with 0.4 *μ*mol kg^−1^ HPPH. After 24 h, hair was removed from the treatment site and from the site that served as unilluminated controls by shaving and depilation with Nair shaver (Carter-Wallace, Inc., New York, NY, USA). Mice were immobilised in specially designed Plexiglas holders without anaesthesia during the course of light delivery. A spot, 1.1 cm in diameter, was exposed to a fluence of 88 or 128 J cm^−2^ at a fluence rate of 14 mW cm^−2^.

The light source consisted of a dye laser (375; Spectra-Physics, Mountain View, CA, USA) pumped by an argon-ion laser (2080; Spectra-Physics), using DCM dye (Exciton, Dayton, OH, USA). The dye laser was tuned to 665 nm. Output from the dye laser was passed through an eight-way beam splitter, and the power of each beam was set individually with Brewster window-type attenuators.

### Reagents

Rabbit antibodies against phosphorylated (Y705)-signal transducers and activator of transcription (STAT)3, phosphorylated p38, PARP, Erk1/2, Bax, and Bcl-xL and mouse monoclonal antibodies against phospho-Erk1/2 were purchased from Cell Signaling Technology (Beverly, MA, USA); rabbit antibodies against STAT3, Bcl-2, cyclin D1, cyclin E1, Cdk2, Cdc25A, p21^Cip1^ and p27^Kip1^p21^Cip1^ and p27^Kip1^ were purchased from Santa Cruz Biotechnology (Santa Cruz, CA, USA) and rabbit antibody against RB was purchased from BD Pharmingen (San Diego, CA, USA). Recombinant human sgp130 was purchased from R&D Systems (Minneapolis, MN, USA). The expression vector and production of Hyper-IL-6 was described previously (Fischer *et al*, 1997; Chattopadhyay *et al*, 2007).

### RNA isolation and reverse transcription-PCR

Total RNA was prepared by using Trizol (Invitrogen, Carlsbad, CA, USA) according to the manufacturer's instructions. In total, 3 *μ*g of total RNA was reverse-transcribed with 200 U of SuperScript III reverse transcriptase (Invitrogen) and 0.5 nmol of oligo (dT)12–18 primer, then polymerase chain reaction (PCR) was performed in 25 *μ*l of reaction solution containing cDNA derived from 50 ng of total RNA, 1 U of *Taq* DNA polymerase (Roche), 2.5 nmol of each dNTP (Roche) and 2 pmol of sense and antisense primer pairs shown below with a DNA thermal cycler. PCR cycles were operated on a regimen of 30 s of denaturation of 94°C; 30 s of primer annealing at the temperature as below and 45 s extension/synthesis at 72°C for 35 cycles. Integrity of the obtained cDNA was tested by amplification of GAPDH transcripts in a 25-cycle PCR reaction. The primers, annealing temperatures and lengths of PCR products are as follows: human (h) IL-6-sense: 5′-CCCCAGTACCCCCAGGAGAAGA-3′, h-IL-6-antisense: 5′-GCTGCGCAGAATGAGATGAGTTGT-3′, annealing temperature 55°C, size 509 bp; h-IL-6R-sense: 5′-CATTGCCATTGTTCTGAGGTTC-3′, h-IL-6R-antisense: 5′-GTGCCACCCAGCCAGCTATC-3′, annealing temperature 63°C, size 280 bp; h-sIL-6R-sense: 5′-GCGACAAGCCTCCCAGGTTC-3′, h-sIL-6R-anti-sense: 5′-GTGCCACCCAGCCAGCTATC-3′, annealing temperature 63°C, size 278 bp; h-oncostatin M receptor (OSMR)-sense 5′-ACCCGGAAGAAAAGGCATTGATT-3′, h-OSMR-antisense: 5′-ACTTGGGGCTTTTGGGGATACTG-3′, annealing temperature 55°C, size 569 bp; gp130-sense: 5′-CATAGTCGTGCCTGTTTGCTTAG-3′, gp130-antisense: 5′-GATCTTCTGGCCGCTCCTC-3′, annealing temperature 55°C, size 527 bp; GAPDH-sense: 5′-TGAACGTCGGTGTGAACGGATTTGGC-3′; GAPDH-antisense: 5′-CATGTAGGCCATGAGGTCCACCAC-3′, annealing temperature 55°C. In all, 20 *μ*l of PCR products were depicted by electrophoresis on 2.0% agarose gels containing ethidium bromide, visualised by UV light and photographed.

### Immunofluorescence

Immunofluorescence staining of HeLa cells was performed with standard techniques using polyclonal rabbit antibodies against the C-terminus of IL-6R*α* of human origin (Santa Cruz, C-20). Cells to be analysed by staining were fixed for 15 min at room temperature in 2% paraformaldehyde, permeabilised in 0.25% Triton X-100 for 30 s, washed twice in phosphate-buffered saline (PBS), and then blocked by incubation in 2.5% bovine serum albumin (BSA) in PBS. Polyclonal rabbit anti-human IL-6 receptor antibody was applied to the slides at a dilution of 1 : 100 and incubated for 60 min at room temperature and then washed three times with PBS. After incubation with fluorescein isothiocyanate (FITC)-conjugated goat anti-rabbit secondary antibody (Sigma, St Louis, MO, USA) at a dilution of 1 : 200 for 60 min at room temperature, samples were washed three times with PBS. DNA was stained by 4,6-diamidino-2-phenylindole (DAPI). The immunofluorescence-labelled cells were then analysed by fluorescence microscopy. Controls of background staining (controls without primary antibody) were processed in parallel.

### Fluorescence-activated cell sorter analysis

Expression of receptor molecules at the cell surface was measured by fluorescence-activated cell sorter (FACS). Briefly, subconfluent monolayer culture cells were released with nonenzymatic dissociation buffer solution (Sigma Chemicals, St Louis, MO, USA), followed by washing two times with sterile PBS, suspension in staining buffer (PBS containing 2% foetal calf serum and 0.09% sodium azide) and staining for 30 min on ice with phycoerythrin-conjugated anti-IL-6R*α* (CD126) antibody (BD Pharmingen). Analysis was performed on a FACSCalibur System using the Cell Quest software (BD Pharmingen).

For cell cycle analysis, cells were harvested, washed and lysed in ice-cold modified Vindelov buffer (0.1% sodium citrate, pH 7.4, 0.02 mg ml^−1^ RNAse A, 0.37% NP-40, 0.5 mg ml^−1^ propidium iodide (PI)) in the dark. The intensity of PI labelling in the isolated nuclei was then measured on a FACScan flow cytometer (Becton Dickinson, Franklin Lakes, NJ, USA).

### Cytokine and soluble receptor measurement

Cell culture supernatant was analysed for cytokine production with a multiplex bead array format using the Bio-Plex/Luminex Instrument. In total, 50 *μ*l per sample was analysed on the Luminex 100 (Bio-Rad, Hercules, CA, USA). For the detection of sIL-6R*α* in supernatants, cells were seeded at a concentration of 10^6^ cells ml^−1^. Supernatants were taken at the indicated interval after treatment. An enzyme-linked immunosorbent assay (ELISA) kit was used (R&D Systems) according to the manufacturer's instructions.

### Western blot analysis

Aliquots of cell extracts containing 20 *μ*g of protein were electrophoresed on 6–12% polyacrylamide gels. The proteins were transferred to nitrocellulose membranes (Schleicher & Schuell, Keene, NH, USA) and reacted with the indicated antibodies. Appropriate peroxidase-conjugated secondary antibodies (ICN Biomedicals, Aurora, OH, USA) were used in PBS containing 0.1% Tween 20 and 5% milk. Immune complexes were visualised by enhanced chemiluminescence reaction (Pierce Biotechnology, Rockford, IL, USA). In each experimental series, X-ray films were exposed for different lengths of time to obtained images with quantitative signals in the linear range of densitometry. Equal loading of samples was verified by Ponceau staining of the proteins immediately after transfer to the nitrocellulose membrane.

### Clonogenic assay

Cells were seeded in 10 cm Petri dishes and left to adhere overnight in regular growth medium as described. 2-[1-Hexyloxyethyl]-2-devinyl pyropheophorbide-a and/or IL-6 cytokines were added as indicated in the text. After PDT treatment, cells were trypsinised and plated in growth medium for colony formation. Two separate experiments were performed using duplicate samples.

### DNA synthesis

DNA synthesis was determined by [^3^H]thymidine incorporation. Briefly, cells were seeded into 24-well culture plates (5 × 10^4^ cells per well). After 48 h, 1 *μ*Ci of [^3^H]thymidine (Amersham Biosciences) was added to each culture and incubation continued for an additional 16 h. Cells were released by trypsin and collected onto paper filter by the cell harvester (Tomtec, Hamden, CT, USA). The amount of incorporated tritium was measured by a scintillation counter (Trilux microbeta, Perkin-Elmer Wallac, Turku, Finland).

### Assessment of tumour response

Orthogonal diameters of tumours were measured once every 2 days with calipers. The tumour volume, *V*, was calculated with the formula *V*=(*lw*^2^/2), where *l* is the longest axis of the tumour, and *w* is the axis perpendicular to *l*. The tumours were monitored until they reached a volume >400 mm^3^, at which time the mice were killed. Regrowing tumours reached the 400 mm^3^ volume within ∼10 days. No tumour regrowth was ever observed later than ∼day 50 and therefore animals were considered cured if they remained tumour free for at least 60 days after PDT.

### Statistical evaluation

The one-tailed Student's *t* test was used for comparison between groups in all of the experiments except for tumour response determinations, with *P*-values of 0.05, representing statistical significance. For tumour response data analysis, hours to event (i.e., to 400 mm^3^ tumour volume) were calculated for each animal by linearly interpolating between the times just before and after this volume was reached, using log (tumour volume) for the calculations; both tumour volume and hours-to-event calculations were performed using Excel (Microsoft, Redmond, WA, USA). Time to progression, defined as a function of time to tumour growth of greater than 400 mm^3^, was determined using a Cox regression model.

## RESULTS

### Conditioned medium from PDT-treated HeLa cells induces macrophages to release sIL-6R*α*

**Figure 1:**
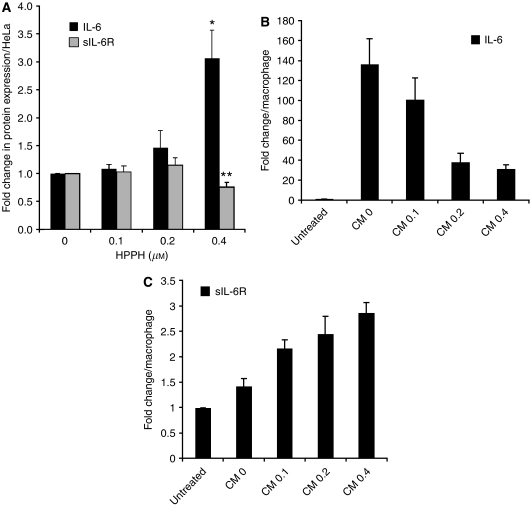
Induction of IL-6 and sIL-6R*α* following PDT. (**A**) HeLa cells were treated with increasing concentrations of HPPH and irradiated with 1 J cm^−2^ light. Conditioned media were collected at 24 h post PDT. (**B**, **C**) Pulmonary macrophages were incubated for 24 h with CM from PDT-treated HeLa cells. The concentrations of IL-6 and sIL-6R*α* in the supernatant culture media were determined by Bio-Plex/Luminex and ELISA, respectively. The values in each experimental series were normalised to the untreated controls and the relative changes determined in three independent experiments were expressed as mean and SD. Values indicated by stars denote difference with *P*<0.05 compared to controls. As reference, the average concentrations for control cultures were as follows: (**A**) IL-6: 180 pg ml^−1^, sIL-6R*α*: 10 pg ml^−1^; (**B**) IL-6 for CM-0 culture: 32 ng ml^−1^; (**C**) sIL6R*α*: 100 pg ml^−1^.

To identify whether PDT might create a milieu that would favour IL-6 trans-signalling, we employed a culture system of HeLa cells and primary human lung macrophages, representing the interacting components of tumour and inflammatory cells. These human cell types were used for the basic assessment of PDT action on IL-6 receptor system because immune reagents were available that permitted the quantification of soluble human IL-6R*α*. First we established that HeLa cells, like other cell types, release increased amounts of IL-6 into the culture medium in response to PDT in a dose-dependent manner (Figure 1A, black bars). The same conditioned medium (CM) did not contain appreciable levels of sIL-6R*α* (Figure 1A, grey bars). Transfer of 24-h CM from control (HPPH – but not light – treated) HeLa cell culture to macrophages stimulated in these the secretion of IL-6 (Figure 1B). In contrast, conditioned media from HeLa cells treated with increasing dose of PDT were progressively less effective in stimulating IL-6 production by macrophages (Figure 1B). The same conditioned media, however, increased up to three-fold the release of sIL-6R*α* (Figure 1C). Given the fact that the *in vivo* tumour milieu contains copious amounts of IL-6 after PDT and macrophages are a component of the PDT-induced host cell infiltrate (Gollnick *et al*, 1997; Henderson *et al*, 2004), these findings argue for a possible role of sIL-6R*α* and IL-6/sIL-6R*α* complex in PDT-treated tumours.

### Interleukin-6 trans-signalling is maintained by PDT-treated tumour cells that have lost their membrane IL-6R*α*

**Figure 2:**
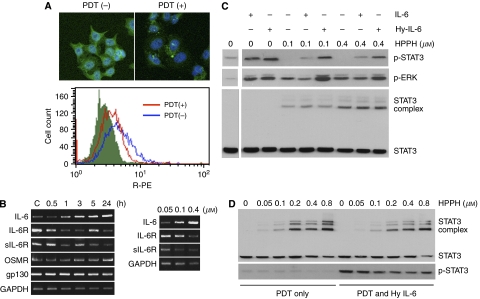
IL-6 trans-signalling in post-PDT cells. (**A**) Interleukin-6R*α* expression in HeLa cells was evaluated by immunofluorescence. Upper panel, cells were seeded onto coverslips. Immediately after treatment with PDT (HPPH 0.4 *μ*M, 1 J cm^−2^), immunostaining was carried out using anti-IL-6R*α* antibody followed by FITC-conjugated secondary antibody. The nucleus was stained with 4,6-diamidino-2-phenylindole, and the labelled cells were observed by fluorescence microscopy. Lower panel, expression of cell surface receptor molecules was measured by FACS analysis. Cells were stained for 30 min on ice with phycoerythrin-conjugated anti-IL-6R*α* (CD126) antibody. (**B**) The effects of PDT on the mRNA levels of IL-6 and IL-6 receptors. HeLa cells were treated with 0.4 *μ*M HPPH and 1 J cm^−2^ of light, and mRNA was extracted at different time period post-PDT (left), or cells were treated with increasing concentrations of HPPH and irradiated with 1 J cm^−2^ of light, and mRNA was extracted at 24 h post-PDT. Reverse transcription–PCR was performed using primers specific for IL-6, IL-6R*α*, sIL-6R*α*, OSMR and gp130. The PCR products were separated on 2% agarose gel and visualised by ethidium bromide staining. Glyceraldehyde 3-phosphate dehydrogenase served as an internal control. (**C**, **D**) Cytokine responsiveness in post-PDT cells. HeLa cells (**C**) or C33A cells (**D**) were treated with increasing concentrations of HPPH, irradiated with 1 J cm^−2^ light. Cytokines (IL-6 100 ng ml^−1^; Hyper-IL-6 160 ng ml^−1^) were added after PDT for 15 min and cells were then extracted. Replicate aliquots of the extracts containing equal amounts of protein were analysed by immunoblotting for the indicated protein.

One of the striking biological consequences of PDT is that surviving cells transiently lose responsiveness to cytokines, such as to IL-6 (Wong *et al*, 2003). Immunofluorescent staining of HeLa cells confirmed that HPPH-PDT caused immediate loss of the cognate membrane-bound IL-6R*α* (Figure 2A). RNA analysis revealed that mRNAs for cognate IL-6R*α* as well as for sIL-6R*α* encoded by an alternatively spliced transcript were retained by PDT-treated cells (Figure 2B), suggesting that post-PDT recovery of the cells could resume IL-6R synthesis from pre-existing mRNA pool. Although not quantitative, the PCR data nevertheless suggested a trend toward reduced mRNA for both IL-6R forms in cells exposed to higher PDT dose (Figure 2B, right panel). The same cells also indicated a PDT-dose and time-dependent increase of IL-6 mRNA in agreement with the known effects including stress-induced expression of IL-6 (Kick *et al*, 1995, 1996; Gollnick *et al*. 1997). The cells also showed that PDT did not cause an appreciable change in mRNA for the co-expressed receptor subunits, OSMR*β* and gp130 (Figure 2B, left panel).

We next addressed the question whether sIL-6R*α* when added together with IL-6 to PDT-treated cells would elicit a response through trans-signalling by engaging gp130. To test this, HeLa cells after exposure to PDT were treated with the designer cytokine Hyper-IL-6 and the response compared to that of IL-6 treatment. The response to cytokine was monitored by the level of STAT3 and ERK phosphorylation (Figure 2C). Photodynamic therapy reduced the STAT3 response to IL-6 by 90%, but that to Hyper-IL-6 only by 30%. The presence of crosslinked STAT3 complexes, which are known to correspond to absorbed PDT dose (Liu *et al*, 2004; Henderson *et al*, 2007), indicated that the individual cultures within each experimental group had received comparable PDT exposure. Taken together, this suggests a preferential PDT-mediated inactivation of IL-6R*α* over gp130, the latter still being able to engage the IL-6/sIL-6R*α* complex.

To demonstrate that Hyper-IL-6 was able to function in PDT-treated cells independently of the membrane IL-6R*α*, the IL-6R-null C33A tumour cell line was subjected to the same PDT treatment as HeLa cells. Signalling in response to Hyper-IL-6 following PDT proved to be similar to that detected in HeLa cells (Figure 2D). Taken together, these data suggest that IL-6 trans-signalling can occur in the post-PDT tumour environment and that it may affect PDT-surviving tumour cells that have lost their IL-6R*α* or cells that are inherently IL-6R*α* negative.

### IL-6 trans-signalling affects cell proliferation following PDT

**Figure 3:**
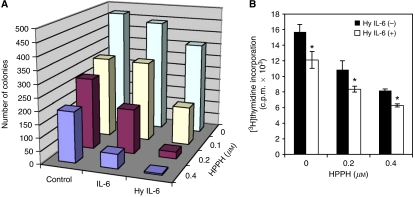
Interleukin-6 and Hyper-IL-6 suppress proliferation of PDT-treated HeLa cells. (**A**) HeLa cells were treated with increasing concentrations of HPPH and exposed to 1 J cm^−2^ of light. After irradiation, cells were plated in 6 cm dishes in the presence or absence of IL-6 cytokines (IL-6 100 ng ml^−1^; Hyper-IL-6 160 ng ml^−1^). After 14 days, colonies were fixed, stained with crystal violet and counted (colonies with >25 cells). The values represent means of a single experiment performed in triplicate, representative of three performed. (**B**) [^3^H]thymidine incorporation into HeLa cells. Post –PDT, cells were treated or not treated with 160 ng ml^−1^ Hyper-IL-6. [^3^H]Thymidine incorporation after 48 h of treatment is plotted as the mean±s.d. of a single experiment performed in triplicate, representative of three independent experiments. ^*^P<0.05, compared with the corresponding value of PDT alone.

The cellular responses to IL-6 stimulation in the presence of functioning signal transduction mechanisms range from growth promotion to growth inhibition (Taga *et al*, 1989; Novick *et al*, 1992). To address the question of potential effects of IL-6 trans-signalling on the proliferation of PDT-surviving tumour cells, we incubated HeLa cells with IL-6, or Hyper-IL-6, following graded PDT doses and assessed survival and proliferation. Clonogenic assay revealed that both IL-6 and Hyper-IL-6 of untreated control cells tended to reduce proliferation, indicating that HeLa cells are subject to some growth control by gp130-derived signals (Figure 3A). Photodynamic therapy reduced in a dose-dependent manner proliferation in the treated cell culture. Choosing PDT conditions that led to a maximal 60% reduction of viable cells, the treatment of the PDT-surviving cell populations with either IL-6 or Hyper-IL-6 showed a further decline of proliferation. Hyper-IL-6 was more effective than IL-6 in suppressing proliferation (Figure 3A) in agreement with the enhanced signalling capability of this factor (Figure 2C). The trends seen in the clonogenic assays were also observed when [^3^H]thymidine incorporation was used as an end point (Figure 3B).

### Photodynamic therapy-dependent growth suppression through IL-6 trans-signalling also occurs in mouse colon26 tumour cells

**Figure 4:**
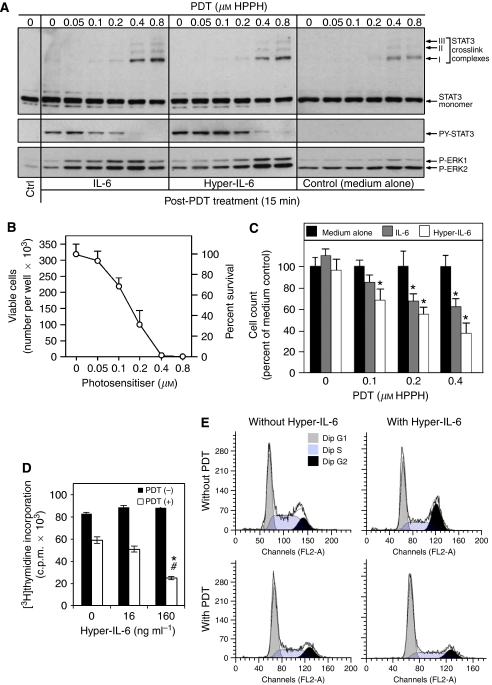
Effect of HPPH-PDT on Colon26 cells. (**A**) PDT dose-dependent crosslinking of STAT3 and loss of STAT3 signalling in Colon26 cells. Colon26 cells were incubated for 4 h with the indicated concentration of HPPH and then exposed to 2 J cm^−2^ light. Immediately following PDT, the cells were treated with IL-6 or Hyper-IL-6 (100 ng ml^−1^ each) for 15 min. Cell extracts were analysed by immunoblotting for the degree of STAT3 crosslinking and treatment-induced changes in phosphorylation of STAT3 and ERK1/2. (**B**) Colon26 cells were treated with HPPH-PDT treated in (**A**) and then incubated in full growth medium for 24 h. The numbers of surviving cells were determined by counting. Mean values+s.d. of four separate experimental series are presented. (**C**) Recovery of proliferation of PDT-treated Colon26 cells. Cells collected after 24 h incubation in (**B**) were diluted in culture medium, plated in replicate cultures wells at <100 viable cells cm^−2^, and cultured for 6 days in full growth medium alone, or medium containing in addition either 100 ng ml^−1^ IL-6 or Hyper-IL-6. The cell counts were determined and expressed relative to the cell counts in the medium control of each series. Mean+s.d. of four separate cultures series in each PDT treatment group are shown. ^*^Denotes *P*<0.05 compared to control. (**D**) Effect of PDT on [^3^H]thymidine incorporation. Colon26 cells were exposed to 1 J cm^−2^, 0.4 *μ*M HPPH-PDT or not, and then treated with various concentrations of Hyper-IL-6. [^3^H]thymidine incorporation was assessed after 48 h of treatment. Shown are the means±s.d. of a single experiment performed in triplicate, representative of three performed. ^*^P<0.001, compared with PDT alone; ^#^*P*<0.001, compared with corresponding non-PDT group. (**E**) PDT effect on cell cycle progression. Colon26 cells were treated as indicated (PDT 0.4 *μ*M HPPH, 1 J cm^−2^; Hyper-IL-6 160 ng ml^−1^) and analysed 72 h post-treatment. Cells were harvested, stained with propidium iodide (PI) and subjected to FACScan flow cytometry. One representative of three independent experiments is shown as histogram.

To apply the findings made in HeLa cells regarding PDT-dependent changes in IL-6 regulation of cell proliferation to an *in vivo* tumour model, we chose the murine colon cell line Colon26. Colon26 cells, like HeLa cells, express endogenous IL-6R system and have a prominent trans-signalling capability in response to Hyper-IL-6 (Figure 4A). Colon26 cells showed an HPPH-PDT dose-dependent photoreaction leading to STAT3 crosslinking (Figure 4A, upper panel), reduction of IL-6 and Hyper-IL-6 responsiveness as evident from the loss of STAT3 phosphorylation (Figure 4A, middle panel) and cell killing (Figure 4B). Colon26 cells also displayed a prominent post-PDT activation of ERK activation (Figure 4A, lower panel) that largely reflected the effect of oxidative stress response. Of note is that the magnitude of this stress-dependent activation of the mitogen-activated protein kinase (MAPK) pathway in Colon26 cells is exceptionally high. The phenomenon of PDT-dependent increase of the growth inhibitory activity of IL-6 and Hyper-IL-6 detected in HeLa cells was also reproduced in Colon26 cells. The magnified effect on proliferation was more prominently evident because Colon26 cells are inherently nonreceptive to IL-6-mediated growth control (Figure 4C). The effect of Hyper-IL-6 on PDT-treated cells was also manifested in a cytokine dose-dependent reduction of DNA synthesis as determined by thymidine incorporation (Figure 4D) that was strikingly similar to that found in HeLa cells (Figure 3B).

The assays employing cell count, thymidine incorporation or clonogenic growth highlighted the reduction of proliferating cells in the PDT- and cytokine-treated cultures. However, the treated cultures also contained viable but nonproliferating cells. Thus, the cell cycle stage distribution of PDT-treated and surviving Colon26 cells cultures was evaluated by flow cytometry. When compared to control cells, HPPH (0.8 *μ*M)-PDT-treated cells showed after 2 days in culture an enhanced fraction of cells in the G1/G0 phase (66 vs 44%) with a concomitant reduction of cells in S and G2 phases. In the presence of Hyper-IL-6 alone, Colon26 cells responded by progressing through S to an accumulation in G2–M phase resulting in a reduction of cells in G1/G0 phase to 42% and an increase of cells in G2 from 22 to 31% (Figure 4E). The specific effects of PDT on cell cycle distribution were maintained even when Colon26 cells were treated with Hyper-IL-6, suggesting a dominant effect of PDT on cell cycle control that is not subject to change by Hyper-IL6. This regulatory phenotype may account, at least in part, for the enhanced suppression of cell proliferation that is observed by the combination of PDT and Hyper-IL-6 treatment.

**Figure 5:**
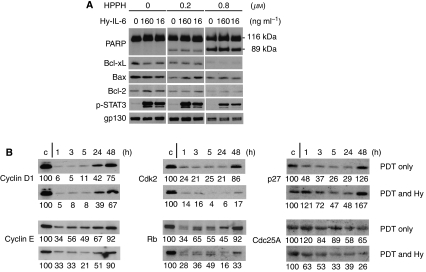
The effects of Hyper-IL-6 on PDT-mediated effects on markers for apoptosis and cell cycle regulation in Colon26 cells. (**A**) Cells were treated with increasing concentrations of HPPH and exposed to 1 J cm^−2^ light. Following illumination, the cells were treated for 4 h with various doses of Hyper-IL-6. Cell lysates were separated on SDS–PAGE and the proteins indicated at the left were identified by immunoblotting. Results are representative of three independent experiments. (**B**) Colon26 cells were subjected to 0.4 *μ*M HPPH and irradiated with 1 J cm^−2^ of light. Immediately after illumination, the cells were treated with or without 160 ng ml^−1^ Hyper-IL-6. Cell lysates collected after the times indicated were analysed by immunoblotting for the proteins marked at the left. *α*-tubulin served as an internal loading control (blots are not shown). Results are representative of three independent experiments. Numbers below lanes represent relative staining intensities or the band relative to the untreated cultures in each series (defined as 100).

Previous studies have suggested that IL-6 stimulation of antiapoptotic or survival effects might assist cells to better withstand PDT-mediated cytotoxicity (Jee *et al*, 2001). Consistent with the above described decreased survival of PDT-treated cells in the presence of IL-6 or Hyper-IL-6, culturing PDT-treated Colon26 cells with Hyper-IL-6 for 4 h was unable to alter the PDT-induced degradation of Bcl-2 and Bcl-xL and execution of caspase-mediated apoptosis (Figure 5A). To extend the assessment to other markers that correlate with growth regulation and, thus, could provide information on the growth suppression by PDT and Hyper-IL-6, we determined expression of representative cell cycle proteins which had been shown to be associated with PDT (Ahmad *et al*, 1998). Western blot analysis (Figure 5B) demonstrated a substantial reduction of cyclin D1, cyclin E, Cdk2, Rb and p27Kip1 during the first 5–24 h post-PDT period as compared to the control. A recovery of expression was detected by 24–48 h. Addition of Hyper-IL-6 to PDT-treated cells did not detectably alter the PDT-regulated expression of the cyclins and Rb, but magnified the loss of Cdk2 and also elicited a reduction of Cdc25A, which was not appreciable affected by PDT alone. In contrast, Hyper-IL-6 was noted to increase p27 that, in the context of post-PDT reaction, amounted to an attenuated suppression by PDT. The more prominent changes in cell cycle controlling proteins found for Hyper-IL-6-treated Colon26 cells would account for the enhanced suppression of proliferation and clonogenicity.

### IL-6 trans-signalling enhances antitumour effects of PDT in mice

Based on the *in vitro* results, we hypothesised that the combination of IL-6 and sIL-6R*α* is present in the tumour milieu and facilitates IL-6 trans-signalling, which in turn has consequences for tumour survival and proliferation. To test this hypothesis, we determined whether IL-6 trans-signalling influences the *in vivo* tumour response to PDT. To this end, we administered sgp130, which selectively inhibits IL-6 trans-signalling, or Hyper-IL-6, which magnifies trans-signalling, to mice carrying Colon26 tumours. sgp130 was given 30 min prior to the start of laser treatment and 3 h after ending PDT treatment, at a time point when we expected an increase of local IL-6 and sIL-6R*α* and its neutralisation to be optimal. The treatment of Hyper-IL-6 was started by an injection before PDT and a follow-up injection 48 h after PDT.

**Figure 6:**
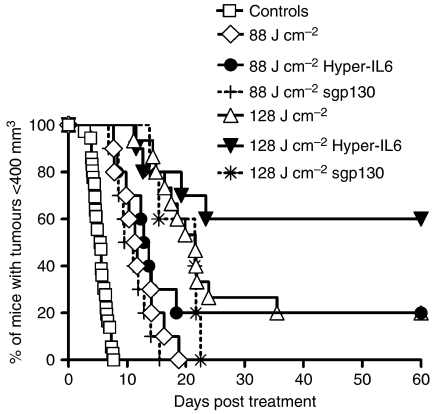
Effect of IL-6/sIL-6R*α* on the outcome of PDT treatment of Colon26 tumour growth. Mice carrying Colon26 tumours were treated with 0.4 *μ*mol kg^−1^ HPPH and irradiated with 88 or 128 J cm^−2^ at 14 mW cm^−2^. Hyper-IL-6 (0.4 *μ*g mouse^−1^) was given, intra peritoneum, immediately and 48 h after PDT. sgp130 (5 *μ*g mouse^−1^) was given i.p. 30 min before and 3 h after PDT. Tumour growth was monitored for 60 days or until tumour reached a volume of 400 mm^3^. For clarity, data for controls have been combined (total of 36 animals); controls included untreated mice, mice with HPPH only and mice with HPPH+Hyper-IL-6 but no light. All experimental groups consisted of 10–15 mice. Results are reported as the percentage of animals with tumours <400 mm^3^. Animals whose tumours had not reached 400 mm^3^ by 60 days were considered cured.

While tumour regrowth after PDT, when it occurred, commenced at the same time in sgp130 and control groups, and proceeded at the same rate, sgp130 reduced the tumour cures observed with PDT alone (Figure 6). In contrast, boosting IL-6 trans-signalling by administration of Hyper-IL-6 resulted in increased tumour control. Similar to the experiments with sgp130, tumour regrowth after PDT, when it occurred, commenced at the same time in Hyper-IL-6 and control groups, and proceeded at the same rate. However, tumour cures were enhanced by Hyper-IL-6 administration from 0 to 20% with 88 J cm^−2^ PDT (median time to growth to 400 mm^3^ 11.5 days and 13.3 days, respectively) and from 20 to 60% with 128 J cm^−2^ PDT (median time to growth to 400 mm^3^ 21.7 days and >60 days, respectively) (Figure 6). While the effects of sgp130 administration can only be considered a trend due to the limited number of animals tested, the effects of Hyper-IL-6 administration were statistically significant (*P*=0.03). Cox regression model analysis revealed an estimated risk ratio of regrowth to 400 mm^3^ and corresponding 95% confidence interval for Hyper-IL-6=absent to Hyper-IL-6=present of 2.3 (1.1, 3.5).

## DISCUSSION

Interleukin-6 membrane receptor (IL-6R*α*) is mainly expressed by hepatocytes, neutrophils, macrophages, lymphocytes and some tumour cells (Scheller *et al*, 2006). Cells, which are deficient or lack IL-6R*α*, can respond to IL-6 when it is associated with a soluble form of the IL-6R*α* (sIL-6R*α*) in a process called trans-signalling (Rose-John and Heinrich, 1994). In this paper, we ask the question whether the PDT-induced loss of IL-6 signalling function could be overcome by trans-signalling mechanisms.

We show the generation of IL-6 by PDT-treated tumour cells as well as by macrophages exposed to medium conditioned by these tumour cells (Figure 1A and B). Interestingly, conditioned media from PDT-treated HeLa cells were progressively less effective in stimulating IL-6 production by macrophages, suggesting a PDT-dependent loss or inactivation of the stimulatory activity factor. At present, the identity of the cytokine-inducing factor with irritant-like activity in conditioned HeLa culture medium has not yet been established. Equally important, we demonstrate that exposure to HeLa cell-CM leads to the generation of sIL-6R*α* by macrophages (Figure 1C), thus furnishing the components necessary for IL-6 trans-signalling. Chalaris *et al* (2007) have recently reported shedding of IL-6R from neutrophils during apoptosis induced by diverse mechanisms such as DNA damage, cytokine withdrawal, Fas stimulation and UV exposure, and implicated caspase-dependent activation of the metalloproteinase ADAM17 as the critical event. Whereas in those experiments neutrophils were exposed directly to the perturbing agent, in our case, the effects were mediated indirectly through transfer of CM. Direct action of PDT on HeLa cells did not generate sIL-6R*α* (Figure 1A), suggesting that PDT did not directly induce receptor shedding although it abolished the membrane-bound IL-6R*α* (Figure 2A) (Wong *et al*, 2003). It is conceivable that the activation of the ADAM17 metalloproteinase is involved in the IL-6R*α* release by HeLa-derived irritants in macrophages; however, the precise molecular mechanism responsible for sIL-6R*α* generation from macrophages remains to be determined.

A major difference in the process by which macrophages and neutrophils release sIL-6R*α* is that the process in macrophages apparently is not tied to apoptosis. Evidence for PDT-dependent generation of regulatory factors or irritants has been detected by Gollnick *et al* (2002) who determined that lysates from PDT-treated tumour cells are able to mature dendritic cells and to stimulate the release of cytokines.

Cells of gp130^+^/IL-6R*α*^−/low^ phenotype, such as endothelial and epithelial cells, in the post-PDT tumour environment can be affected by the presence of IL-6 via trans-signalling mechanisms (Figures 2 and 3). The cells' response to IL-6 is mediated through the JAK/STAT signal transduction pathway, where STAT3, after phosphorylation, plays a central role in transmitting signals from the membrane to the nucleus (Hirano *et al*, 2000). Rakemann *et al* (1999) have shown that Hyper-IL-6 is a potent activator of STAT3-dependent gene transcription. In cells of the B-cell lineage and in plasmacytoma/myeloma cells, this facilitates transmission of prosurvival signals, the enhancement of proliferation, differentiation and inhibition of apoptosis by induction of the antiapoptotic protein Bcl-xL; in hepatocytes, it leads to maturation and enhancement of liver regeneration (Schwarze and Hawley, 1995; Minami *et al*, 1996; Hirano *et al*, 2000). Overexpression of IL-6 has been shown to either enhance or decrease sensitivity of tumour cells to PDT-induced cytotoxicity by modulating apoptotic threshold (Jee *et al*, 2001; Usuda *et al*, 2001). Conversely, IL-6 cytokine family members oncostatin M and leukaemia inhibitory factor (LIF) have been shown to suppress epithelial growth (Grant *et al*, 2001).

In the present study it is evident that in PDT-treated cells, IL-6 exerts antiproliferative activity, similar to the suppression of proliferation in epithelial cells by OSM (Chattopadhyay *et al*, 2007). In HeLa cells, IL-6 and even more so Hyper-IL-6 alone reduced cell proliferation, and enhanced the antiproliferative effects of PDT (Figure 3). In murine Colon26 cells, the growth of which does not appear to be influenced by gp130 stimulation via Hyper-IL-6 alone, the latter also induced prolonged growth arrest after PDT (Figure 4). Studies by others have shown that PDT disturbs cell cycle progression at the G1/S transition, which leads to growth arrest and apoptosis (Ahmad *et al*, 1998; Haywood-Small *et al*, 2006). The presence of Hyper-IL-6 enhanced and prolonged the suppression of especially Cdk2. Our results further showed that this decrease in Cdk2 is correlated with hypophosphorylation of Rb (Figure 5B), which has been recognised to inhibit proliferation through regulation of E2F-responsive genes (Ahmad *et al*, 1999). It was shown earlier that PDT induced p21^WAF1/Cip1^, which resulted in a pronounced decrease in cyclin D1, Cdk2 expression and to a lesser extent, cyclin E and Cdk6 (Ahmad *et al*, 1998). Our studies disclose an increased p27^Kip1^ and a decreased Cdc25A expression by Hyper-IL-6 treatment in post-PDT cells (Figure 4), suggesting that cdk2/cyclin E might be the molecular target involved in IL-6 trans-signalling-mediated cell cycle arrest. Supportive evidences from previous studies showed that IL-6-type cytokines were able to inhibit Cdk2 activity and DNA synthesis by accumulation of p27^Kip1^ protein (Klausen *et al*, 2000). Furthermore, it has been reported that IL-6 was able to induce degradation of Cdc25A by STAT3 activation, which would form a repressor complex with the Rb tumour suppressor to occupy the Cdc25A promoter and block its induction (Bernardi *et al*, 2000; Barre *et al*, 2005).

Considering the results of this study, one can construct the following scenario. The post-PDT tumour milieu contains abundant amounts of IL-6 and other inflammatory mediators (Gollnick *et al*, 2003) to which surviving cells within the treatment field do not respond directly because of PDT-induced degradation and inactivation of their membrane-bound IL-6R*α*. Leucocytes respond to the tissue injury, enter the tumour environment, generate sIL-6R*α* as well as IL-6 and thus allow the transmission of IL-6 signals in surviving tumour cells. Moreover, this also allows stromal cells, which have a gp130^+^/IL-6R*α*^−/low^ phenotype, to respond to IL-6. While we may be able to credit the antiproliferative effects of IL-6 signalling for enhancing tumour control, broader effects on the inflammatory response may well also be involved. We and others have shown earlier that the inflammatory response contributes to tumour control by PDT (Korbelik, 1996; Henderson *et al*, 2004), and we have evidence that it contributes to antitumour immunity (unpublished). Interleukin-6 is increasingly recognised as critical for the successful resolution of inflammation and as a major immunological switch from innate to acquired immunity (Jones, 2005). Interleukin-6 signalling may also contribute to antitumour immunity by inducing T_H_-17 cells in collaboration with TGF*β*, while suppressing TGF*β*-driven Fox3 expression, which induces immunosuppressive regulatory T cells (Bettelli *et al*, 2007). T_H_-17 cells produce IL-17, which coordinates tissue inflammation by inducing inflammatory cytokines, chemokines and matrix metalloproteinases capable of mediating tissue infiltration and destruction.

In summary, this study has for the first time identified IL-6 trans-signalling as a mechanism orchestrating the tissue and host response to PDT. As in preclinical models, clinical application of PDT results in local and systemic inflammation, which can be characterised by elevated cytokine levels, including IL-6. In severe cases, this inflammatory response can constitute dose-limiting toxicity and require pharmacological intervention (Yom *et al*, 2003). It is therefore of the utmost importance to fully understand the mechanisms governing all aspects of the host response to PDT and the consequences of this response for tumour control. Here we provide evidence for the biological and possibly clinical relevance of the IL-6 signalling pathways for the antitumour activity of PDT.
